# Cystathionine β-synthase is inhibited by epinephrine and norepinephrine over-secretion via NF-κB activation in stress-induced hyperhomocysteinemia

**DOI:** 10.1038/s41598-026-52669-3

**Published:** 2026-05-19

**Authors:** Ling Zhang, Shuqing Wu, Fang Xie, Xue Wang, Zhaowei Sun, Yun Zhao, Lingjia Qian

**Affiliations:** Beijing Institute of Basic Medical Sciences, No. 27 Taiping Road, Beijing, 100850 China

**Keywords:** Stress, Homocysteine, Epinephrine/norepinephrine, IL-6, Cystathionine β-synthase, Biochemistry, Cell biology, Molecular biology, Physiology

## Abstract

Stress can elevate plasma homocysteine (Hcy) levels, a key factor in the development of various diseases, leading to hyperhomocysteinemia (HHcy). The enzyme Cystathionine β-synthase (CBS) functions in the trans-sulfuration pathway, which links the methionine cycle to glutathione synthesis. This pathway is essential for the metabolism of homocysteine. However, the precise mechanism by which stress regulates hepatic CBS expression remains unclear. The present study aimed to elucidate the molecular mechanism by which CBS expression is regulated in the livers of restraint-stressed rats. Our results showed that stress-induced over-secretion of epinephrine and norepinephrine (E/NE) activated the β-adrenergic receptor, leading to elevated serum IL-6 levels. Treatment of rat primary hepatocytes with IL-6 for 1 h suppressed both CBS activity and *Cbs* mRNA expression, concomitant with an enhancement of Sp3-mediated transcriptional repression. Furthermore, we found that IL-6 activates NF-κB via the tyrosine phosphorylation pathway, which in turn potentiates Sp3-mediated repression of *Cbs* transcription. These findings suggest that E/NE contributes to stress-induced HHcy by inhibiting *Cbs* transcription through the upregulation of Sp3, and that the IL-6/NF-κB axis plays a critical role in the dysregulation of Hcy metabolism.

**Supplementary Information:**

The online version contains supplementary material available at 10.1038/s41598-026-52669-3.

## Introduction

Stress is a psychophysiological response triggered by an imbalance between environmental demands and the body’s coping ability^1,2^. It activates the hypothalamic-pituitary-adrenal (HPA) axis and the sympathetic/adrenal medullary system, leading to the secretion of neuroendocrine hormones, including epinephrine/norepinephrine (E/NE), and glucocorticoids^3,4^. These hormones affect various organs, resulting in changes across the nervous, endocrine, immune, and other systems^5,6^.

Previous studies have shown that stress can induce elevated plasma homocysteine (Hcy) levels in rats^7–9^. Hcy is a cytotoxic sulfur-containing amino acid, an intermediate in methionine metabolism. Elevated plasma Hcy levels, known as hyperhomocysteinemia (HHcy), are considered a significant risk factor for various diseases and are closely linked to abnormal changes in neuroendocrine hormones during stress^10^.

Hcy metabolism involves two main pathways: the re-methylation pathway, which produces methionine via methionine synthase (MS) and betaine-homocysteine-methyltransferase (BHMT), and the trans-sulfuration pathway, which produces cysteine through the action of cystathionine β-synthase (CBS) and cystathionine γ-lyase (CSE)^11,12^. CBS, the only enzyme for the catabolic removal of Hcy in mammals, is the rate-limiting step in the trans-sulfuration pathway. Studies indicated that stress-induced HHcy is primarily caused by a reduction in CBS activity in the liver, which plays a crucial regulatory role between the methionine cycle and trans-sulfuration of Hcy metabolism. Inhibition of trans-sulfuration metabolism in the liver leads to Hcy accumulation in the body^13^.

CBS, an enzyme encoded by the *Cbs* gene, is regulated by the transcription factors Sp1 and Sp3, with NF-Y acting as a cofactor that synergistically enhances their activity^14^. Sp1/Sp3 belong to the Specificity Protein transcription factor family of ubiquitously expressed factors, which activate or repress transcription of many genes in response to physiological and pathological stimuli. Sp1/Sp3 have been suggested as potential trans-activators for the human *CBS-1b* promoter, bind to the same DNA elements with similar affinity, while Sp1 positively regulates *Cbs* transcription, Sp3 acts as a negative regulator^15–17^. Previous studies^18^ have shown that stress-induced activation of the HPA axis can suppress hepatic *Cbs* transcription by upregulating Sp3 expression via glucocorticoid receptors (GR), and inducing HHcy. However, the role of the sympathetic-adrenal-medullary axis—simultaneously activated by stress—and its secreted catecholamines in the development of HHcy remains unclear, particularly whether it enhances Sp3-mediated transcriptional repression through immune-inflammatory signaling pathways.

While the individual roles of stress hormones, IL-6, NF-κB, and Sp3 in inflammation and gene regulation are well-recognized^19–22^, the precise molecular cascade connecting adrenergic signaling to hepatic *Cbs* suppression remains incompletely defined. Crucially, it is unknown whether and how the key inflammatory mediator IL-6, induced by stress, orchestrates the interaction between NF-κB and Sp3 to regulate *Cbs* transcription in hepatocytes. This study aims to clarify how catecholamine hormones regulate CBS expression in the rat liver under stress. Our results found that in stress-induced HHcy, E/NE inhibits *Cbs* transcription by upregulating Sp3, with the IL-6/NF-κB pathway playing a key role in this process. Importantly, we provided novel evidence that IL-6 stimulation promoted a direct protein-protein interaction between NF-κB and Sp3 in primary hepatocytes, which underpinned the transcriptional repression of the *Cbs* gene.

## Results

### Elevated E/NE and IL-6 inhibited CBS enzyme activity and transcriptional level in stress-induced HHcy

**Fig. 1:**
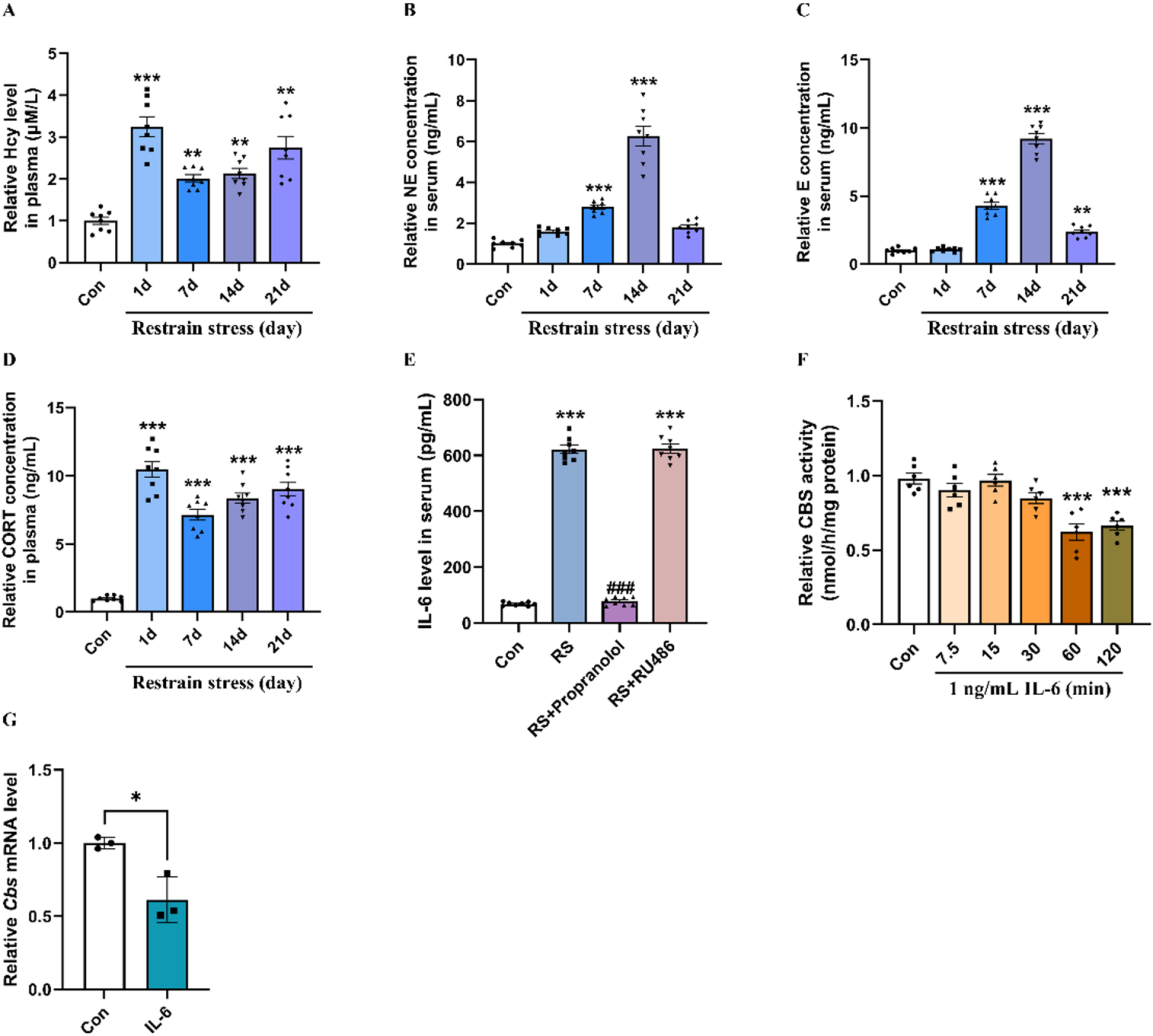
The role of E/NE and IL-6 on CBS enzyme activity and transcriptional level in stress-induced HHcy. (**A**) Effect of restraint stress on Hcy levels in rat plasma, *n* = 8/group. (**B**–**D**) Effect of restraint stress on the concentration of epinephrine (E), norepinephrine (NE) and corticosterone (CORT) in rat serum/plasma, *n* = 8/group. (**E**) Effect of propranolol (β-adrenergic receptor antagonist) and RU486 (glucocorticoid receptor antagonist) on IL-6 level in rat serum with 14 days of restraint stress, *n* = 8/group. (**F**, **G**) Effect of 1ng/mL IL-6 on CBS activity for 7.5, 15, 30, 60 and 120 min (*n* = 6) and *Cbs* mRNA level for 60 min (*n* = 3) in rat primary hepatocytes. *p < 0.05, **p < 0.01, ***p < 0.001 vs. Con, ^###^ *p* < 0.001 vs. RS.

RS significantly elevated plasma Hcy levels in rats across different stress groups compared to the control group (Fig. 1A). The highest level was observed in the acute stress group (1 day), which was 3.2 times higher than that in the control group, while chronic stress caused plasma Hcy levels to gradually increase by 2–3 times with increasing stress intensity, indicating that both acute and chronic restraint stress could induce HHcy. Additionally, RS significantly increased serum E/NE levels (Fig. 1B–C), with 14-day RS leading to peak serum E/NE levels at 6.3 and 9.2 times respectively (*p* < 0.001) compared to controls. The plasma CORT level in the acute stress group (1 day) was approximately 10‑fold higher than that in the control group, and in the chronic RS group, the CORT level increased by 7–10 times with increasing stress intensity (Fig. 1D, *p* < 0.001). Furthermore, 14 days of RS significantly elevated serum IL-6 levels. Serum IL-6 levels in control rats were less than 100 pg/mL (0.1 ng/mL), and in rats subjected to 14 days of restraint stress, levels ranged from 500 to 700 pg/mL (0.5 to 0.7 ng/mL) (Fig. 1E, *p* < 0.001). Intraperitoneal injection of propranolol (10 mg/kg weight), a β-adrenergic receptor antagonist, reversed the IL-6 increase in RS rats (less than 100pg/mL, *p* < 0.001), while the glucocorticoid receptor antagonist mifepristone (RU486, 30 mg/kg weight) had no effect (Fig. 1E), suggesting that elevated serum IL-6 in stressed rats may result from β-adrenergic receptor activation by E/NE. CBS, a key enzyme in hepatic trans-sulfuration metabolism, plays a critical role in stress-induced HHcy. Previous studies have shown RS markedly inhibited *Cbs* transcription in rat liver^18^. Here, based on the pathological results of serum IL-6 concentration in RS rats, hepatocytes treated with 1 ng/ml IL-6 for 60 and 120 min exhibited a 40% decrease in CBS activity (*p* < 0.001) and inhibited *Cbs* transcription (*p* = 0.0145) compared to control (Fig. 1F–G). These results suggest that the neuroendocrine network of stress may induce HHcy through IL-6-mediated transcriptional regulation of CBS.

### IL-6 inhibited CBS transcription via affecting NF-κB and Sp1/Sp3 levels and their interactions in rat hepatocytes

**Fig. 2:**
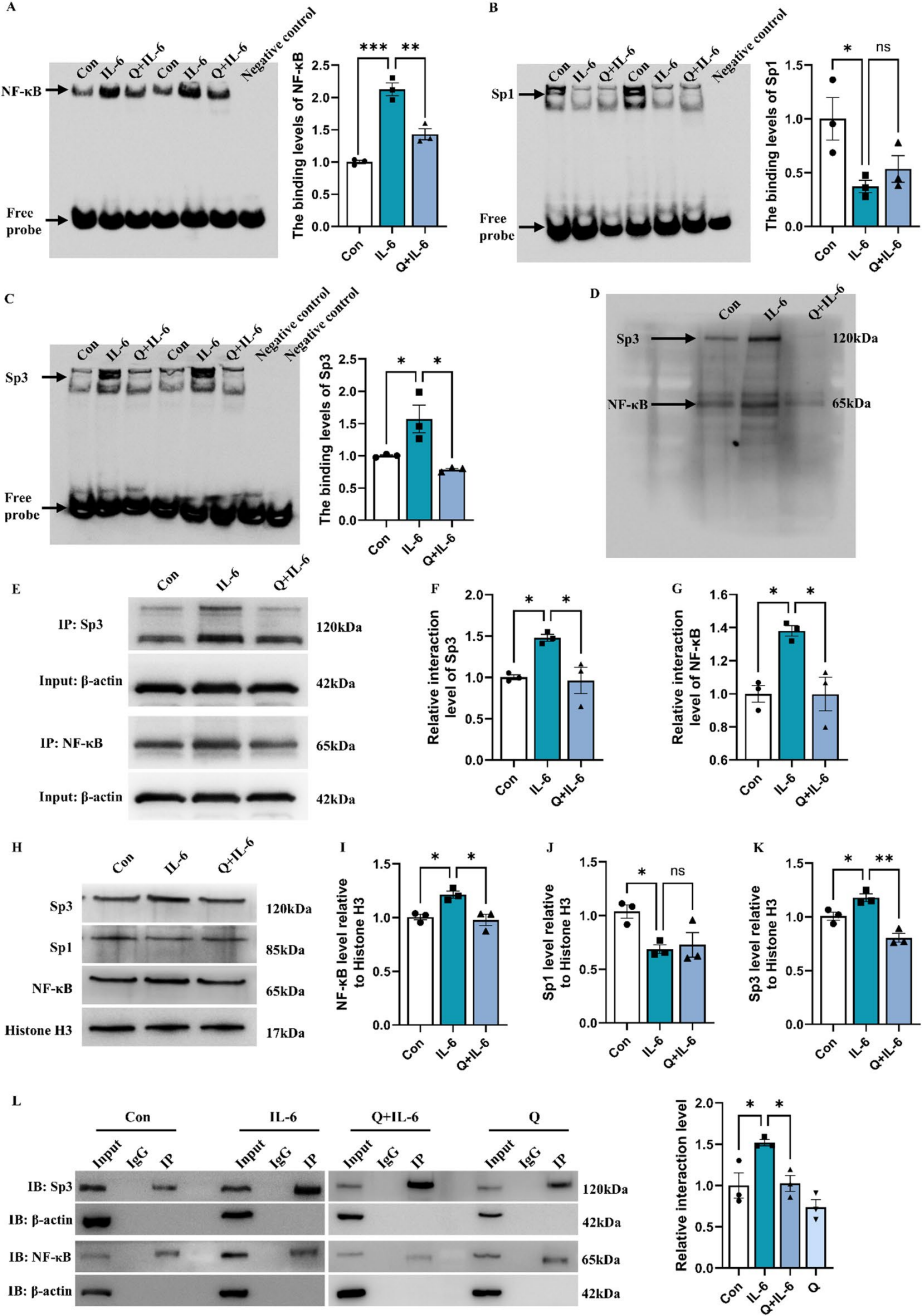
The effect of IL-6 on NF-κB and Sp1/Sp3 levels and interactions in rat primary hepatocytes. (**A**–**C**) EMSA was performed to detect the binding levels of NF-κB and Sp1/Sp3 to *Cbs* in nucleus of rat primary hepatocytes with the treatment of 1 ng/ml IL-6 for 1 h and 150 µM quercetin for 3 h. Negative control: no protein extract for DNA to bind, thus there was only an unshifted probe band. (**D**) Western blotting detected tyrosine phosphorylation protein level of Sp3 and NF-κB in the nucleus of rat hepatocytes under IL-6 and quercetin treatment. (**E**–**G**) Co-immunoprecipitation (Co-IP) was performed to demonstrate directly that IL-6 promotes tyrosine phosphorylation of Sp3 and NF-κB in the nucleus of rat primary hepatocytes. (**H**–**K**) Effect of IL-6 and quercetin on NF-κB and Sp1/Sp3 level in nucleus of rat primary hepatocytes via western blotting. (**L**) The interaction of NF-κB and Sp3 in nucleus of rat primary hepatocytes was showed by Co-immunoprecipitation (Co-IP). *n* = 3. *p < 0.05, **p < 0.01, ***p < 0.001.

The transcription factors Sp1 and Sp3 directly regulate *Cbs* transcription by competing for the same binding site. After 60 min of 1 ng/ml IL-6 treatment, the binding levels of NF-κB to *Cbs* increased by 2.13 times (*p* = 0.0001), the binding level of Sp3 to *Cbs* increased by 1.57 times (*p* = 0.0413), while the level of Sp1 at the same binding site as Sp3 decreased by 60% (*p* = 0.0415). And the effects were reversed by the treatment of 150 µM quercetin for 3 h, a tyrosine phosphorylation inhibitor, which restored the binding levels of NF-κB (*p* = 0.0018) and Sp3(*p* = 0.0103) without affecting Sp1 (*p* = 0.6979) (Fig. 2A–C). IL-6 also enhanced the tyrosine phosphorylation level of NF-κB and Sp3 (Fig. 2D). Moreover, Co-IP experiments were performed to further determine that both NF-κB and Sp3 were detected in the tyrosine‑phosphorylated immune-precipitates, and their levels were markedly increased by about 1.5 times upon IL-6 stimulation, and this effect was reversed by the treatment of quercetin (Fig. 2E–G). These results directly demonstrate that IL-6 promotes tyrosine phosphorylation of Sp3 and NF-κB. Similarly, IL-6 treatment resulted in a 1.2-fold increase in the nuclear content of NF-κB (*p* = 0.0210) and Sp3 (*p* = 0.0486), while a 30% decrease in Sp1 level (*p* = 0.0453), and quercetin inhibited this effect (Fig. 2H–K). These findings suggested that IL-6 inhibited CBS activity by activating NF-κB and Sp3 via tyrosine phosphorylation. Immunoprecipitation further revealed that IL-6 enhanced the interaction between NF-κB and Sp3 in hepatocytes, evidenced by a 1.5-fold increase in Sp3 level in the NF-κB protein complex (*p* = 0.0307), which was inhibited by quercetin (Fig. 2L), and this interaction was tyrosine phosphorylation-dependent.

### The activation of Sp3 mediated by NF-κB resulted in the inhibition of *Cbs* transcription in response to IL-6 stimulation

**Fig. 3:**
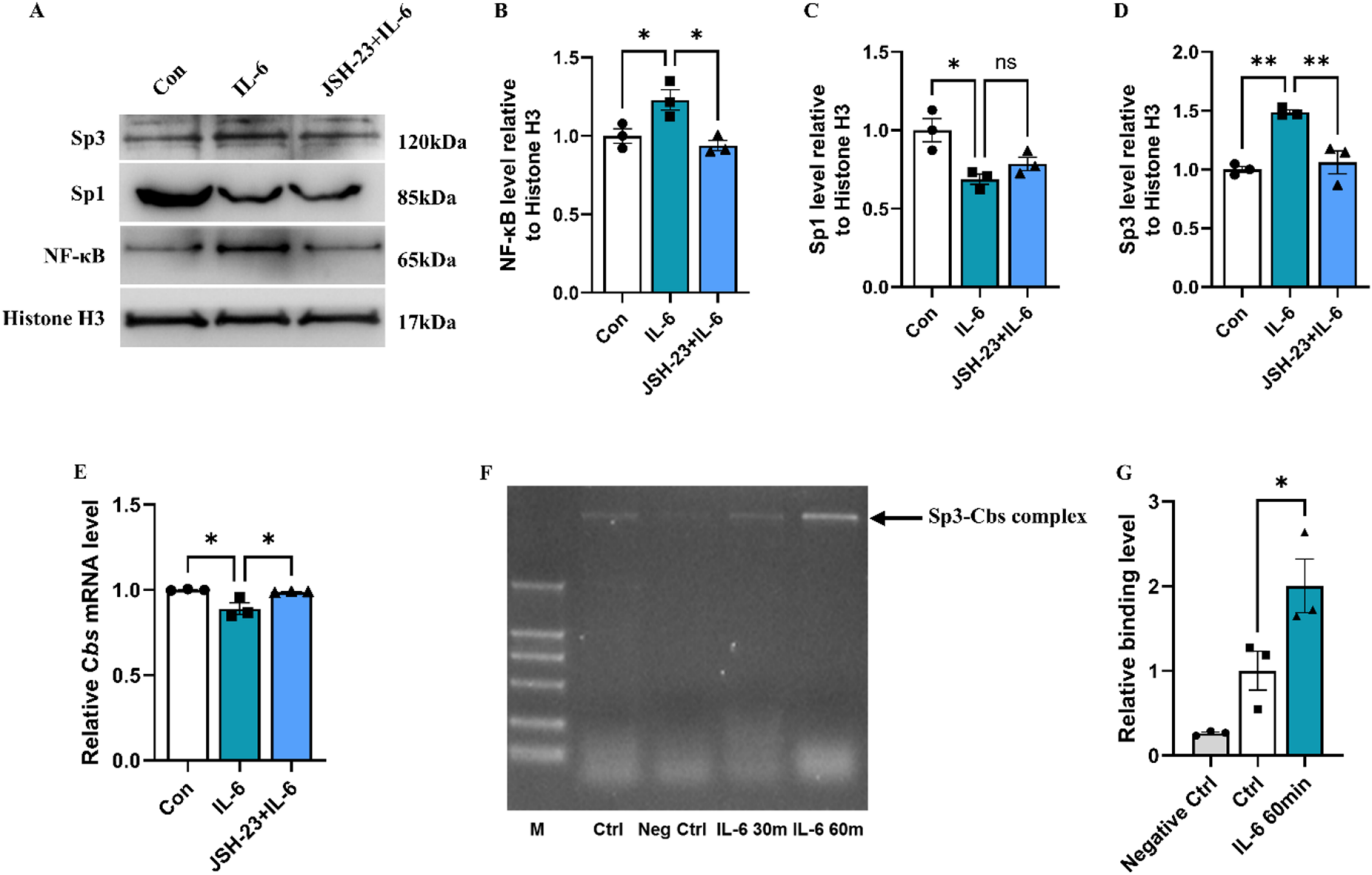
The activation of Sp3 mediated by NF-κB resulted in the inhibition of *Cbs* transcription in the nucleus of rat hepatocytes. (**A**–**D**) Western blotting showed that treatment with 30 µΜ JSH-23 for 1 h reversed the nuclear levels of NF-κB and Sp3 in rat primary hepatocytes with IL-6 stimulation. (**E**) Effect of 30 µΜ JSH-23 on *Cbs* mRNA levels in rat primary hepatocytes. (**F**, **G**) ChIP showed the regulation of Sp3 on *Cbs* transcription in IL-6-treated hepatocytes. *n* = 3. *p < 0.05, **p < 0.01.

To investigate the regulatory relationship between the interacting transcription factors NF-κB and Sp3, the NF-κB inhibitor JSH-23 was used. Western blot analysis revealed that the increasement of NF-κB nuclear content (*p* = 0.0406) induced by IL-6 was antagonized by 30 µΜ JSH-23 treatment for 1 h (23.7% inhibition, *p* = 0.0147) (Fig. 3A–B), confirming that JSH-23 effectively inhibited NF-κB level in the hepatocyte nucleus. Furthermore, elevated Sp3 level induced by IL-6 (*p* = 0.0029) returned to control levels (decreased by 28.7%, *p* = 0.0056) after JSH-23 treatment, while Sp1 level remained unchanged after JSH-23 treatment (Fig. 3A & C–D), suggesting that NF-κB regulated Sp3 content in the hepatocyte nucleus in response to IL-6 intervention. Additionally, IL-6-induced downregulation of *Cbs* transcription (decreased by 11%, *p* = 0.0169) was significantly increased after JSH-23 treatment (*p* = 0.0299) (Fig. 3E), and ChIP results demonstrated that Sp3 binding to the *Cbs* promoter was enhanced by 2-fold (*p* = 0.0466) after 60 min of IL-6 treatment (Fig. 3F–G). In conclusion, NF-κB-mediated activation of Sp3 plays a key role in the transcriptional regulation of *Cbs* under IL-6 stimulation.

## Discussion

Studies^7,8,23^ have shown that various external factors can trigger a strong stress response, leading to elevated Hcy levels. Hcy, an essential sulfur-containing amino acid in the methionine cycle, is metabolized to methionine or cysteine with the help of cofactors such as folate, vitamin B_6_, and vitamin B_12_^24,25^. Our previous studies^26^ indicated that stress reduces the expression of key enzymes MS, CBS, CSE in the Hcy metabolic pathway, resulting in HHcy, a known risk factor for several diseases. CBS is the only enzyme in mammals used for catabolic removal of Hcy, catalyzing a rate-limiting step in Hcy metabolism via the trans-sulfuration pathway^12,27^. The previous results^18^ have confirmed that the development of HHcy induced by RS stress was mainly derived from a reduction of CBS activity in the liver, which was accompanied by a significant decrease in its mRNA level. However, the molecular mechanisms regulating CBS expression warrant further investigation.

Stress can induce prolonged activation of the sympathetic-adrenal-medullary and hypothalamic-pituitary-adrenal (HPA) axes, leading to excessive secretion of catecholamines (CAs) and glucocorticoids (GCs), which affect multiple organs and trigger changes in neuroendocrine, metabolic, and immune systems^28–30^. Previous study^18^ has shown that RS stress activates the HPA axis and induces HHcy through glucocorticoid-mediated inhibition of CBS. In this study, we found that RS stress activates the sympathetic-adrenal-medullary system, increasing the secretion of epinephrine (E) and norepinephrine (NE), and significantly raising serum IL-6 levels via β-adrenergic receptor activation. Furthermore, IL-6 intervention in primary hepatocytes for 1 h downregulated CBS activity and mRNA level, suggesting that IL-6 mediates the regulation of Hcy metabolism through the neuroendocrine-immune network in stressed organisms, affecting *Cbs* transcription and contributing to HHcy. However, the underlying mechanism requires further exploration.

Studies have identified the upstream cis-response elements involved in *Cbs* transcriptional regulation, including the GC box, reverse CAAT box, and E box, with corresponding trans-acting factors Sp1/Sp3, nuclear factor (NF)-Y, and upstream stimulator 1 (USF-1), respectively^12,14,31^. Sp1 and Sp3 directly regulate *Cbs* transcription, and their ratio determines the direction of *Cbs* regulation of target genes. Sp1 activates *Cbs* transcription, while Sp3 competes with Sp1 for the same DNA binding site, inhibiting Sp1-mediated activation and negatively regulating *Cbs* gene expression^32–35^. Our results showed a significant increase in NF-κB and Sp3 content and binding level to *Cbs* in hepatocyte nucleus with IL-6 treatment, accompanied by a decrease in Sp1 level. This suggests that high Sp3 levels in the nucleus competitively inhibit Sp1, thereby hindering Sp1 activation. Sp3 was the key point of variations in *Cbs* transcription caused by stress.

To further investigate how IL-6 regulates NF-κB and Sp3 to influence CBS transcription, this study used 150 µM quercetin for inhibition of tyrosinase and confirmed that IL-6 activated Sp3 by inducing NF-κB and Sp3 tyrosine phosphorylation, which enhanced protein-protein interactions. ChIP and PCR analysis revealed an increased presence of Sp3-specific fragments in hepatocytes following IL-6 treatment, indicating that IL-6 promotes Sp3 binding to the *Cbs* promoter and induces the down-regulation of *Cbs* transcription. Similarly, Ge et al. demonstrated Sp1/Sp3 binding to the *Cbs-1b* promoter in HepG2 cells regulates CBS transcriptional activity, influenced by Sp1/Sp3 phosphorylation status^36^. Wu et al. found that increased Sp1 phosphorylation contributes to reduced CBS transcription and activity in ischemia/reperfusion rat kidneys^37^. Evidence shows that an increased Sp1/Sp3 ratio often correlates with enhanced gene transcription, as most Sp3 isoforms inhibit Sp1-driven transcriptional activation^33,38,39^. However, growing research suggests that Sp3 regulates gene expression dualistically, both activating and inhibiting transcription, with phosphorylation playing a key role^40–42^. Several studies have shown that Sp1/Sp3 share structural and functional similarities in cancer cells, with their synergistic changes contributing to cancer progression^14,43,44^. Additionally, Zhu et al. found that Sp3 modulates CSTB promoter binding level in human hepatocytes, thereby enhancing gene transcription^21^. Sp3 has also been shown to induce inflammasome activation in microglia by binding its promoter, enhancing GRIK1 transcription^22^. These findings suggest that Sp1/Sp3-mediated transcriptional regulation varies across cell and animal models, as well as with the phosphorylation status of transcription factors. Furthermore, following JSH-23 inhibition of NF-κB nuclear content, a reversal in IL-6-induced Sp3 levels and significant restoration of *Cbs* mRNA was observed, indicating that NF-κB is a key mediator in Sp3 activation by IL-6. NF-κB signaling plays a crucial role in cytopathological processes by regulating gene expression^45^. Carver et al. found that NF-κB activates Sp3 binding, which inhibits Sp1-mediated target gene expression upon exposure to inflammatory stimuli^46^. Jiang et al. observed NF-κB pathway activation and downregulated CBS expression in a lipopolysaccharide (LPS) induced inflammation cell model^47,48^. However, Yuan et al. demonstrated that NF-κB upregulates CBS expression in irritable bowel syndrome rats, contributing to visceral hypersensitivity^49^. Another study shown that an epigenetic regulation might be involved in the development of rats’ gastric hypersensitivity by enhancing NF-κB-mediated *Cbs* gene expression^50^. Zhu et al. also found that elevated plasma norepinephrine (NE) activated β_2_-adrenergic receptors, enhancing colonic CBS expression and thereby inducing visceral hypersensitivity^51^. Therefore, either positive or negative regulation of *Cbs* by NF-κB were depend on different disease devolvement, which observed repression of CBS was specific to IL-6–driven, stress–related conditions in hepatocytes in this study.

Overall, our findings demonstrate that stress-induced over-secretion of epinephrine and norepinephrine elevates IL-6 levels in the serum of stressed rats via β-adrenergic receptor activation. IL-6 treatment inhibits CBS activity and *Cbs* mRNA levels in rat hepatocytes, enhancing Sp3 regulation of *Cbs* transcription. IL-6 further activates NF-κB through a tyrosine phosphorylation pathway, mediating transcriptional repression of *Cbs* by Sp3. These results suggest that the IL-6/NF-κB pathway plays a crucial role in Hcy metabolism disorder. Here, the analysis revealed that exposure of primary hepatocytes to 1 ng/mL IL-6 for different time, a significant decline in CBS activity began at 60 min. Consequently, investigating the upstream signaling events—including the tyrosine phosphorylation of NF-κB and Sp3, their enhanced nuclear translocation and interaction—at this phenotypically relevant time point provides a coherent link between pathway activation and functional outcome. However, we acknowledge that it does not encompass the potential longer-term regulatory effects of sustained IL-6 exposure. Sustained IL-6 exposure over several hours or days may involve additional, distinct layers of regulation^52–54^(e.g. secondary transcriptional feedback, epigenetic modifications, or changes in protein stability) that were not assessed in the current study. This represents a valuable direction for future research to obtain a more comprehensive understanding of the chronic regulation of hepatic CBS under inflammatory stress. In addition, all in vivo experiments were conducted exclusively in male rats. This design choice, while common for initial mechanistic studies to reduce variability, precludes the generalization of our conclusions to females. Given the well-documented sex differences in stress responses^55,56^, IL-6 signaling^57,58^, and homocysteine metabolism^59^, our findings may represent a male-specific pathway. The potential influence of sex hormones, such as estrogen which can modulate NF-κB level and inflammatory gene expression^60–62^, or testosterone, which may affect adrenergic receptor sensitivity^63,64^, on the proposed IL-6/NF-κB/Sp3 axis remains an open and critical question for future research. Investigating whether the observed mechanism operates similarly, is attenuated, or is fundamentally different in females will be essential for a complete understanding of stress-induced HHcy pathophysiology across sexes.

## Conclusion

**Fig. 4:**
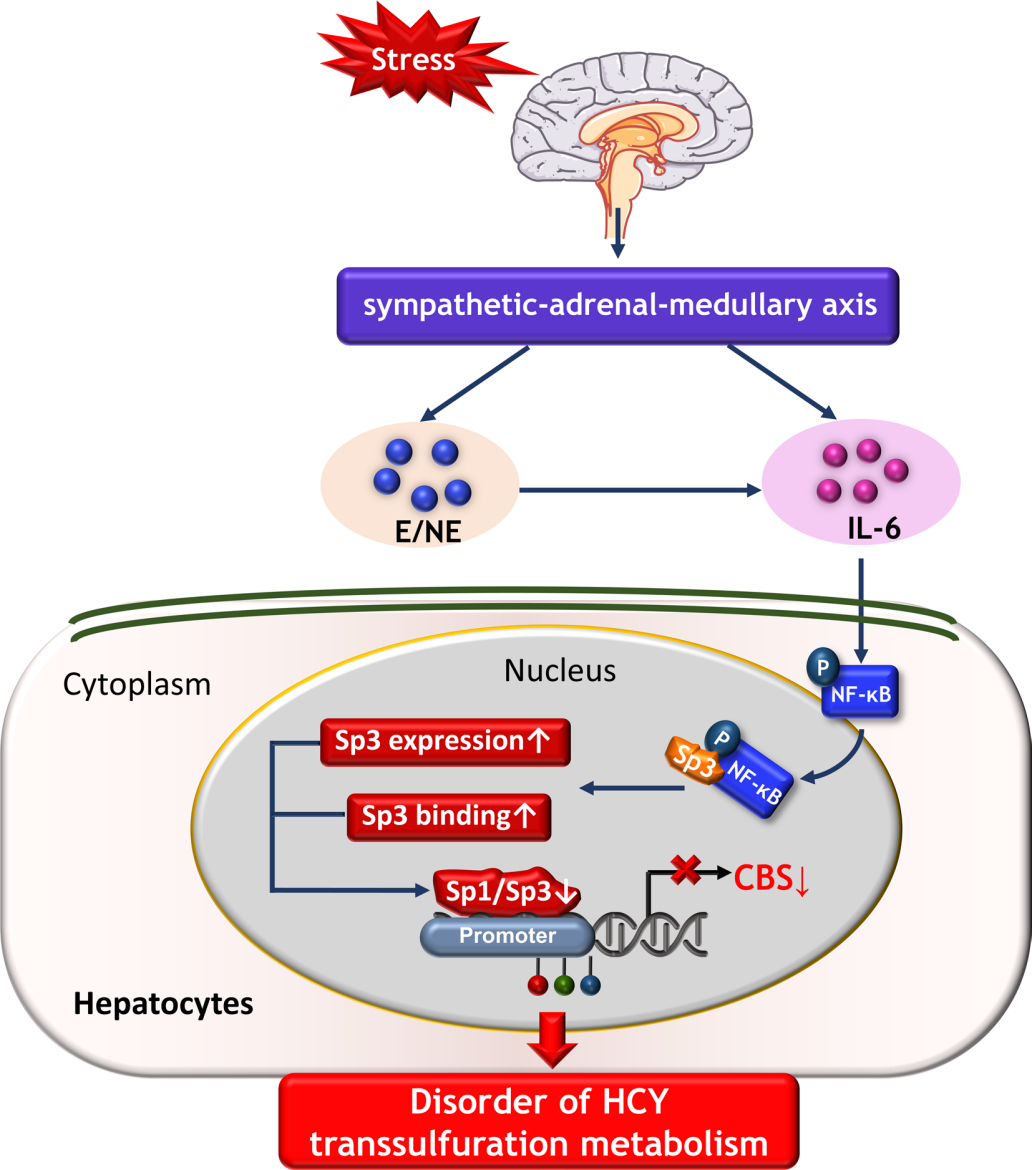
Schematic diagram of the signaling cascade triggered by restraint stress leading to HHcy.

In summary, the study indicated that RS stress inhibits hepatic CBS in rats by elevating E/NE levels, leading to HHcy. As schematically summarized in Fig. 4, IL-6 activates Sp3 through NF-κB, enhancing its protein-protein interaction with Sp3, with tyrosine phosphorylation being essential for this process. Sp3 activation strengthens its binding to the *Cbs* promoter, inhibits Sp1-dependent *Cbs* transcription, and reduces *Cbs* mRNA levels in hepatocytes, thereby decreasing enzyme activity. This study highlights the pivotal role of the IL-6-NF-κB-Sp3 axis in regulating stress-induced HHcy within the neuroendocrine-immune network.

Restraint stress triggers the over-secretion of E/NE from the sympathetic-adrenal-medullary axis, which results in elevated serum IL-6 levels. IL-6 activates NF-κB in hepatocytes via tyrosine phosphorylation. Activated NF-κB enhances the tyrosine phosphorylation and functional activation of the transcriptional repressor Sp3. Sp3 subsequently binds to the *Cbs* promoter, antagonizing Sp1-mediated transactivation, thereby suppressing *Cbs* transcription. The reduction in CBS expression leads to decreased enzyme activity and the accumulation of Hcy, resulting in HHcy. This figure highlights the central role of the IL-6–NF-κB–Sp3 axis in linking the neuroendocrine stress response with metabolic dysregulation of homocysteine metabolism.

## Materials and methods

### Experimental animals and Rat model of chronic restraint stress (RS)

The animal model of restraint stress was established as described previously^65^. To avoid the influence of hormone cycle fluctuations, only male rats were selected for the experiments. Eight-week-old male Sprague-Dawley (SD) rats (purchased from Beijing Sprague-Ford Laboratory Animals) were randomly divided into control group and four groups with different stress intensities (1, 7,14 and 21 days), with 8 rats in each group. To further investigate the mechanism by which stress influenced IL-6 levels, subsets of stressed rats received intraperitoneal injections of either Mifepristone (MedchemExpress, HY-B05738, dissolved in physiological saline at 10 mg/kg weight) or RU486 (Sigma, M8046, dissolved in peanut oil at 30 mg/kg weight). Stress rats were placed individually in a custom-made well-ventilated hard plastic restraint tube for 6 h per day (9 am to 3 pm) for 21 consecutive days. All experimental animals were cessation of food and water intake during this period, and were free to eat and drink normally the rest of the time and maintained in a temperature-controlled environment with a 12 / 12 h light–dark cycle.

24 h after the last stress exposure, rats were anesthetized via intraperitoneal injection of freshly prepared 2.5% tribromoethanol (Meilunbio, MA1613-1) at a dose of 0.8-1 mL/100 g weight. The solution was prepared on the day of the experiment, stored in the dark at 4 ℃, and administered using aseptic techniques. Successful anesthesia was confirmed by the absence of the pinched-toe reflex. Under deep anesthesia, terminal blood sampling was performed via cardiac puncture, which served as the humane endpoint for the experiment, and animals were promptly sacrificed by cervical dislocation afterward. Death was confirmed by observing permanent cessation of respiration, absence of cardiac activity, and dilated, fixed pupils. Following confirmation of death, liver tissue samples were rapidly collected and stored at -80 ℃ for further analysis. All experiments were approved by the Ethics Committee of the Institutional Animal Care and Use Committee of the Academy of Military Medical Sciences (Permit No: IACUC-DWZX-2022-640) and were in accordance with the National Research Council’s Guide for the Care and Use of Laboratory Animals (8th edition) and the ARRIVE guidelines from NC3Rs. We made all efforts to minimize animal suffering.

### Culture and intervention of primary hepatocytes

Hepatocytes were isolated from male rat livers by a classical two-step collagenase perfusion technique^66^. Seven-week-old male Sprague-Dawley (SD) rats (purchased from Beijing Sprague-Ford Laboratory Animals) were anesthetized with 0.8 mL/100 g of tribromoethanol, and physiological responses were monitored to confirm deep anesthesia. The rats were then positioned on an operating table, and a midline incision was made in the chest using sterile surgical scissors to expose the liver and major blood vessels. A perfusion catheter was inserted through the portal vein, followed by the injection of 15–20 mL of 37 °C PBS to chelate calcium and wash out blood. Following that, collagenase ((Liberase™ TM Research Grade, Sigma-Aldrich, 05401127001) was perfused to the liver in order to dissociate extracellular matrix. Finally, the liver was dissected and high viability hepatocytes were purified by Percoll-based density separation. The hepatocytes were counted and plated on collagen-coated cell culture plates/wells with Dulbecco’s modified eagle medium (DMEM, Sigma, St. Louis, MO, USA) supplemented with 5% fetal bovine serum (FBS) (Gibco, 10099141 C) and then further cultured in William’s E media (WE) (Gibco, 12551-032) supplemented with 1% L-Glutamine (100 U/mL, ) and 1% penicillin–streptomycin (100 U/mL, Solarbio, Beijing, China) at 37℃ with 5% CO_2_. After 24 h of hepatocytes were cultured in serum-free medium, they were incubated with 150 µM quercetin for 3 h, 30 µΜ JSH-23 for 1 h, and 1 ng/mL IL-6 for different time points (when the experiment was needed).

### Determination of serum/plasma epinephrine (E), norepinephrine (NE), corticosterone (CORT), and IL-6 levels

The levels of E/NE, CORT and IL-6 in serum/plasma of rats were detected separately using the kit: Epinephrine ELISA Kit (Elabscience, E-EL-0045), Norepinephrine ELISA Kit (Elabscience, E-EL-0047), Rat Corticosterone ELISA kit (Sango biotech, D731285-0096) and Rat IL-6 ELISA kit (Sigma-Aldrich, RAB0311).

### Determination of plasma homocysteine (Hcy)

Blood samples were collected from rats into EDTA-coated tubes and immediately centrifuged at 3,000 × g for 15 min at 4 ℃ to obtain plasma. Hcy levels in rat plasma were detected by high-performance liquid chromatography (HPLC) with fluorescence detection, following previously described methods^67,68^. Briefly, an aliquot of 240 µL of plasma was mixed with 60 µL of 2.5 mM N-acetylcysteine in distilled water as the internal standard. The Hcy-mixed disulfides or protein-bound Hcy in the plasma were treated with TCEP in order to reduce thiols and release them from plasma proteins. Proteins were precipitated by adding 0.6 M cold perchloric acid containing 1 M EDTA. The samples were vortexed vigorously, left at room temperature for 10 min, and then centrifuged at 2,000 × g for 10 min at 4 ℃. Subsequently, the thiol compounds in the samples were derivatized with a thiol-specific fluorogenic reagent, SBD-F (pH 9.5). The mixture was incubated in a water bath at 60 ℃ for 60 min in the dark to ensure complete derivatization of thiol compounds. After incubation, the reaction was terminated by placing the samples on ice, and the resulting solution was transferred to amber HPLC vials for analysis. Hcy in samples was analyzed using a HPLC system consisting of a Waters 2695 Liquid chromatograph (LC2695, Waters Corporation, USA) and a Waters 2475 fluorescence detector (LC2475, Waters Corporation, USA) at excitation wavelength 390 nm and emission wavelength 470 nm. Separation was performed using a reversed-phase column (Symmetry Shield™ RP18, 3.9 × 150 mm, 5 μm) at an isocratic concentration (0.08 M NaAc, 1% methanol in water) at a flow rate of 0.8 mL/min for 15 min. Hcy peaks were identified by comparing their retention times with those of authentic Hcy standards. Quantification was performed using the internal standard method. A calibration curve was constructed by plotting the peak area ratio (Hcy/acetylcysteine) against the concentration of Hcy in the standard solutions. The Hcy concentration in each plasma sample was calculated from this calibration curve and expressed as µmol/L.

### Determination of cystathionine β-synthase (CBS) activity

Rat hepatocytes CBS enzyme activity was determined using a Cystathionine beta Synthase Assay Kit (Abcam, ab241043). Briefly, 500 µL of CBS assay buffer was added to 10 mg of cell pellet and homogenized on ice using a Dounce homogenizer, and then centrifuged at 10,000 × g for 15 min at 4 °C and the supernatant was collected to enzyme assays. Measuring fluorescence immediately at Ex/Em = 368/460 nm in kinetic mode for 1 h at 37 °C. CBS enzyme activity is expressed as nmol/h/mg protein.

### Analysis of *Cbs* mRNA level

Total RNA was extracted from hepatocytes using Trizol reagent (#93289, Sigma-Aldrich, USA) and reverse transcribed to cDNA following the instructions of the reverse transcription kit (#G490, Abmole, Canada). qPCR was performed using TB Green Premix Ex TaqTM (DRR820A, Takara, Japan) with β-Actin as the reference gene and the analysis was performed using the 2-ΔΔCq method. The sequences of the primers used are shown in Table 1.

**Table 1:** List of primers for PCR.

Gene	Forward	Reverse
*Cbs*	GATGAGTATGGAGAAGGTGGA	GAATCGAATCTGGCGTTGG
β-actin	CTTCCTGGGTATGGAATCCT	TCT TTACGGATGTCAACGTC

### Hepatocyte nuclear protein isolation and extraction

Hepatocytes were collected and nuclear proteins were isolated according to the instructions of the Nuclear Protein and Cytoplasmic Protein Extraction Kit (P0027, Beyotime, China). Briefly, 200 µL of cytoplasmic protein extraction reagent A with PMSF was added for every 20 µL of cell pellet, and placed on ice for 10 min, then 10 µL of reagent B was added following by ice bath for 1 min, and then centrifuges at 12,000–16,000 × g for 5 min at 4 ℃. Aspirated the residual supernatant completely, and 50 µL of nuclear protein extraction reagent supplemented with PMSF was added to resuspend the pellet, incubated on a roller drum at 4 ℃ for 30 min, and then centrifuged at 14,000 ×g for 10 min at 4 ℃. The supernatant was the nuclear extract and stored at -80 ℃ until analysis.

### Western blotting

Nuclear protein concentration was determined using a BCA Protein Assay Kit (TianGen, #W9924, China). The samples were subjected to SDS–PAGE and subsequently transferred onto a PVDF membrane (Bio-Rad, USA). Afterward, the membrane was blocked with 5% milk for 1 h. The membrane was incubated with primary antibodies (Sp1, Sp3 and NF-κB, Cell Signaling Technology, USA; Phospho-tyrosine, Immunoway, China), diluted in the ratio 1:1000 and Histone H3 diluted in the ratio 1:5000 overnight at 4 ℃. After washing with TBST, the membranes were incubated with anti-rabbit IgG labeled with horseradish peroxidase (#RS0002, Immunoway, China) diluted in the ratio 1:2000 at room temperature for 2 h. A chemiluminescence imager was used for the analysis.

### Electrophoretic Mobility Shift Assay (EMSA)

To detect the binding level of NF-κB and Sp1/Sp3 to *Cbs* DNA sequences, the Light Shift Chemiluminescent EMSA Kit (Thermo Fisher Scientific, 20148, USA) was used for detection of the protein–DNA complexes. The complementary single-stranded oligonucleotides labeled with biotin at the 5’ end (synthesized and 5’ labeled by Sangon, Shanghai, China) are listed in Table 2. To generate the double-stranded DNA probe for EMSA, equimolar amounts of the forward and reverse oligonucleotides were mixed in annealing buffer (10 mM Tris-HCl, pH 8.0, 50 mM NaCl, 1 mM EDTA). The mixture was heated to 95 ℃ for 5 min and then gradually cooled to room temperature over 1 h to allow for proper annealing. The resulting double-stranded probe was stored at -20 ℃ until use. Nuclear extracted proteins were incubated in the reaction buffer on ice for 10 min, followed by the addition of the biotin-labeled probes for another 20 min at room temperature. For super-shift assays, antibodies of Sp1(CST, #80784) or Sp3 (CST, #3900) were added to the mixture before adding the probe to clarify the specific identity of the binding protein. All reaction products were separated by 6% native polyacrylamide gel electrophoresis, transferred to a positively charged nylon membrane, and visualized by detecting the chemiluminescent signal of biotin-labeled probes.

**Table 2:** List of oligonucleotide sequences used for electrophoretic mobility shift assay (EMSA) probe preparation.

Gene	Forward	Reverse
*Cbs*-NF-κB	TTCCTGGGAATTCCGGTTAG	CTAACCGGAATTCCCAGGAA
*Cbs*-Sp1/Sp3	GCACCACCACGCCCATTTTAA	TTAAAATGGGCGTGGTGGTGC

All oligonucleotides were synthesized with a 5’ biotin modification.

### Co-immunoprecipitation (Co-IP)

Protein-protein interactions between NF-κB and Sp3 were assessed by co-immunoprecipitation. In brief, rat primary hepatocytes were lysed on ice for 30 min using ice-cold cell lysis buffer (CST, 9803 S, USA) supplemented with 1 mM PMSF. The crude lysate was then centrifuged at 14,000 × g for 10 min at 4 ℃ to remove cellular debris. The supernatant containing total protein was collected, and its concentration was determined. To reduce non-specific binding, the protein supernatant was pre-cleared by incubation with protein A/G agarose beads (CST,37478 S, USA) at 4 ℃ for 1 h, followed by centrifugation at 2,500 × g for 5 min. The pre-cleared supernatant was aliquoted into three groups: Input (total protein control), IgG (isotype control), and IP (specific immunoprecipitation). For the IP groups, the supernatant was incubated overnight at 4 ℃ with gentle rotation with specific primary antibodies: mouse anti-NF-κB antibody (1:100, CST, 6956T, USA) for precipitating NF-κB complexes. Parallel IgG control groups were incubated with species-matched normal IgG (mouse IgG_1_ for NF-κB IP, 5415, CST) at the same concentration. Following the overnight incubation, protein A/G agarose beads were added to each IP and IgG sample to capture the antibody-protein complexes, with continued incubation at 4 ℃ for 3 h. The beads were then collected by brief centrifugation at 2,500 × g for 30 s and washed five times with cold cell lysis buffer. Finally, the immunoprecipitated proteins were eluted from the beads by boiling in 1× SDS-PAGE loading buffer for 10 min. The eluted proteins (IP and IgG samples) along with the Input sample were subsequently analyzed by SDS-PAGE and Western blotting.

The tyrosine phosphorylation of Sp3 and NF-κB were detected by Co-IP. Nuclear extracts were prepared from rat primary hepatocytes under IL-6 and quercetin treatment. The rest of the procedure is the same as above. Here, tyrosine‑phosphorylated proteins were immunoprecipitated using an anti‑phospho-tyrosine antibody (1:50, CST, #96215, USA). The immune-precipitates were then subjected to western blotting with antibodies specific for Sp3 or NF‑κB.

### Chromatin immunoprecipitation (ChIP)

ChIP was performed using the SimpleChIP^®^ Plus Sonication Chromatin IP Kit (#56383, Cell Signaling Technology). In brief, the cells were treated with 1% formaldehyde to cross-link the proteins and DNA for 20 min. The Cell pellet was resuspended with a lysis buffer after centrifugation and homogenized into a single-cell suspension. Sonication was used to produce the chromatin fragments of 200–1000 bp. Next, the resulting supernatants were incubated overnight at 4 ℃ with the Sp3 Polyclonal Antibody (1:100, YT2916, CST, USA) on the gentle rotation. Subsequently, the supernatants were mixed with ChIP-Grade Protein G magnetic beads for 2 h at 4 ℃ and subjected to magnetic separation to isolate the DNA-protein complexes. After removing the supernatant, the complexes were extracted and the cross-links were reversed. Spin columns were used to purify the DNA, which was then subjected to PCR analysis and amplified fragments of Sp3 (458 bp) were analyzed in TAE buffer using a 2% agarose gel. The primers used for *Cbs* detection are provided in Table 1.

### Statistical analyses

All experiments were performed independently at least three times. All the statistical analyses were conducted with GraphPad Prism 9.0 software. Prior to parametric testing, all datasets were assessed for normality using the D’Agostino-Pearson omnibus test. For comparisons between the means of two independent groups, a two-tailed unpaired Student’s t-test was employed when the normality assumption was satisfied. If the normality test failed, the nonparametric Mann-Whitney U test was used instead. For comparisons among more than two groups, a one-way analysis of variance (ANOVA) was performed after verifying both normality and the homogeneity of variances across groups. Homogeneity of variance was tested using Bartlett’s test. When these assumptions were met, Tukey’s honestly significant difference (HSD) post hoc test was applied for multiple comparisons. In cases where variances were significantly different (heteroscedasticity), Welch’s ANOVA followed by Dunnett’s T3 multiple comparisons test was used to ensure robustness. Data are shown as mean ± standard error (SEM), and *p* < 0.05 was considered a significant result.

## Supplementary Information

Below is the link to the electronic supplementary material.

Supplementary Material 1

## Data Availability

The data will be made available from the corresponding author on request.

## Abbreviations

BHMT: Betaine-homocysteine-methyltransferase
CBS: Cystathionine β-synthase
CORT: Corticosterone
CSE: Cystathionine γ-lyase
E/NE: Epinephrine/norepinephrine
GR: Glucocorticoid receptor
Hcy: Homocysteine
HHcy: Hyperhomocysteinemia
HPA: Hypothalamic-pituitary-adrenal
IL-6: Interleukin-6
MS: Methionine synthase
NF-κB: Nuclear factor κB
RS: Restraint stress
