# Supplementary figures and images for: Cystathionine β-synthase is inhibited by epinephrine and norepinephrine over-secretion via NF-κB activation in stress-induced hyperhomocysteinemia
Source: Sci Rep. 2026 May 19;16:22772. doi: 10.1038/s41598-026-52669-3 (PMC13385765; doi:10.1038/s41598-026-52669-3)

Figure 2A

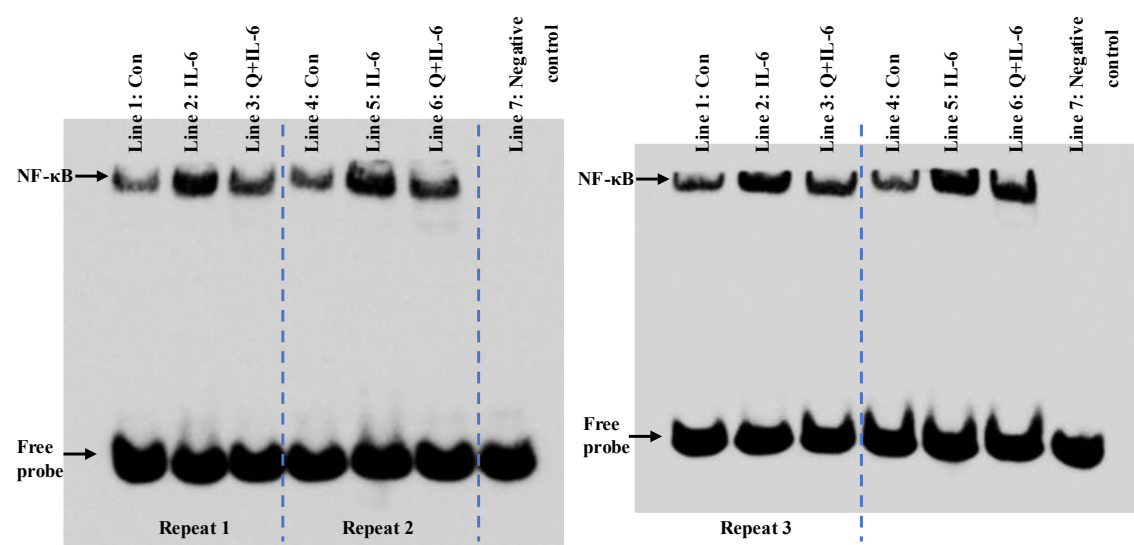

Figure 2B

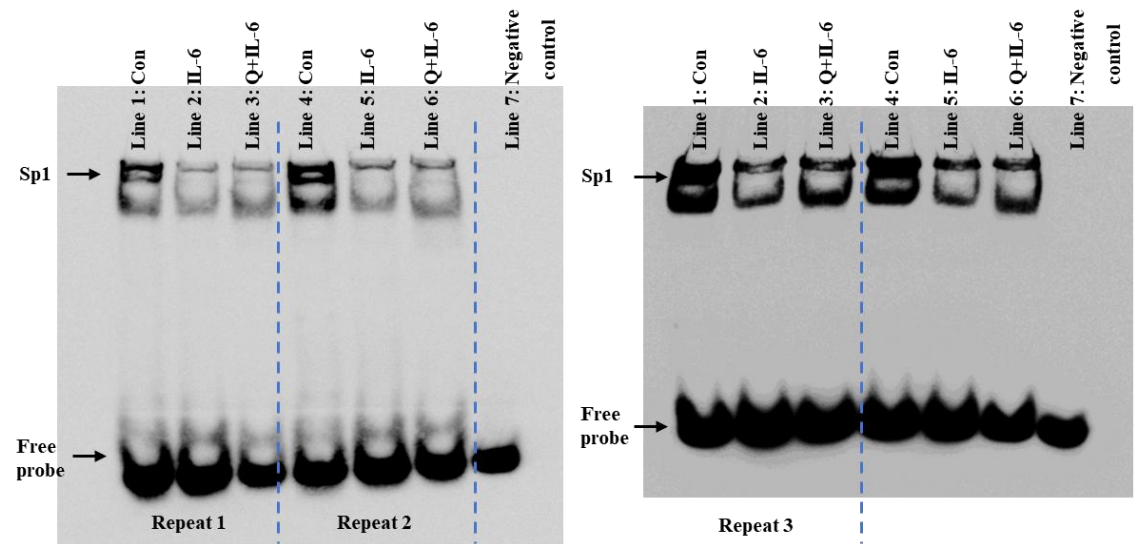

Figure 2C

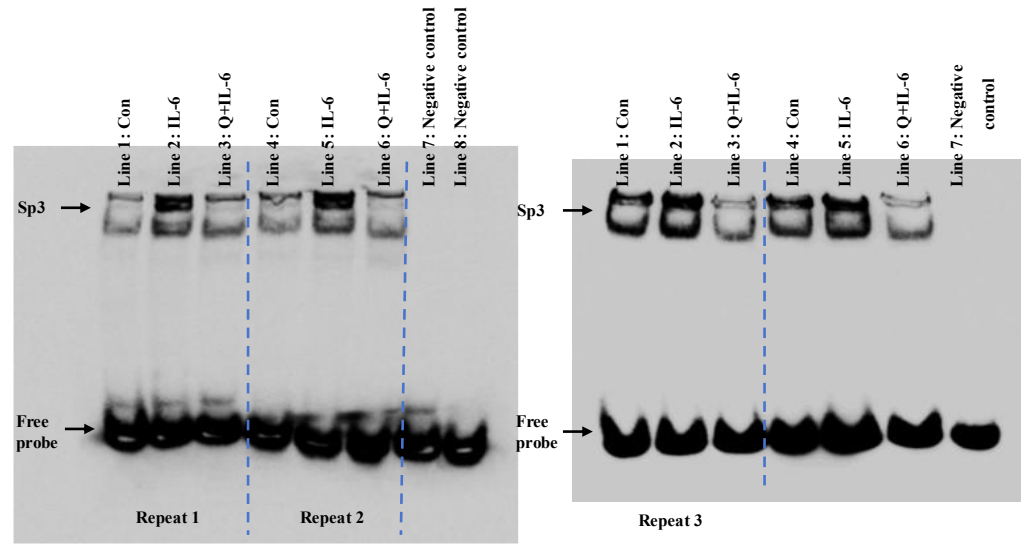

Figure 2D

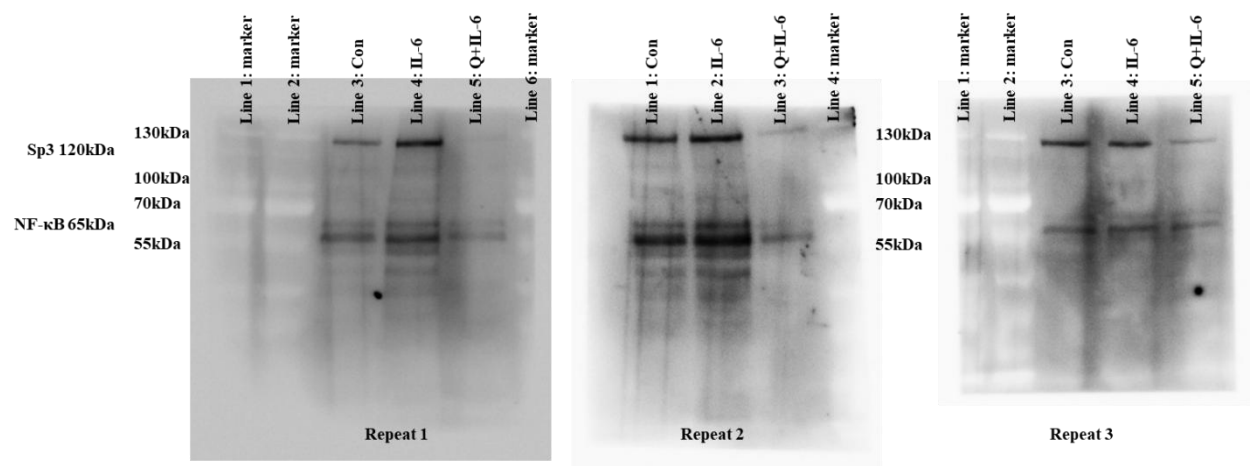

Figure 2E

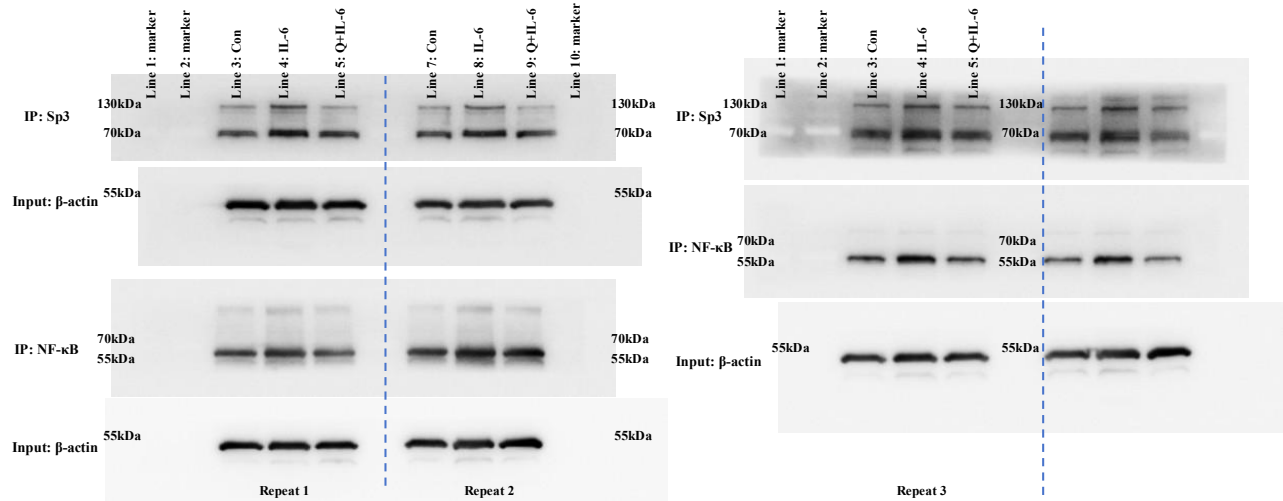

Figure 2H

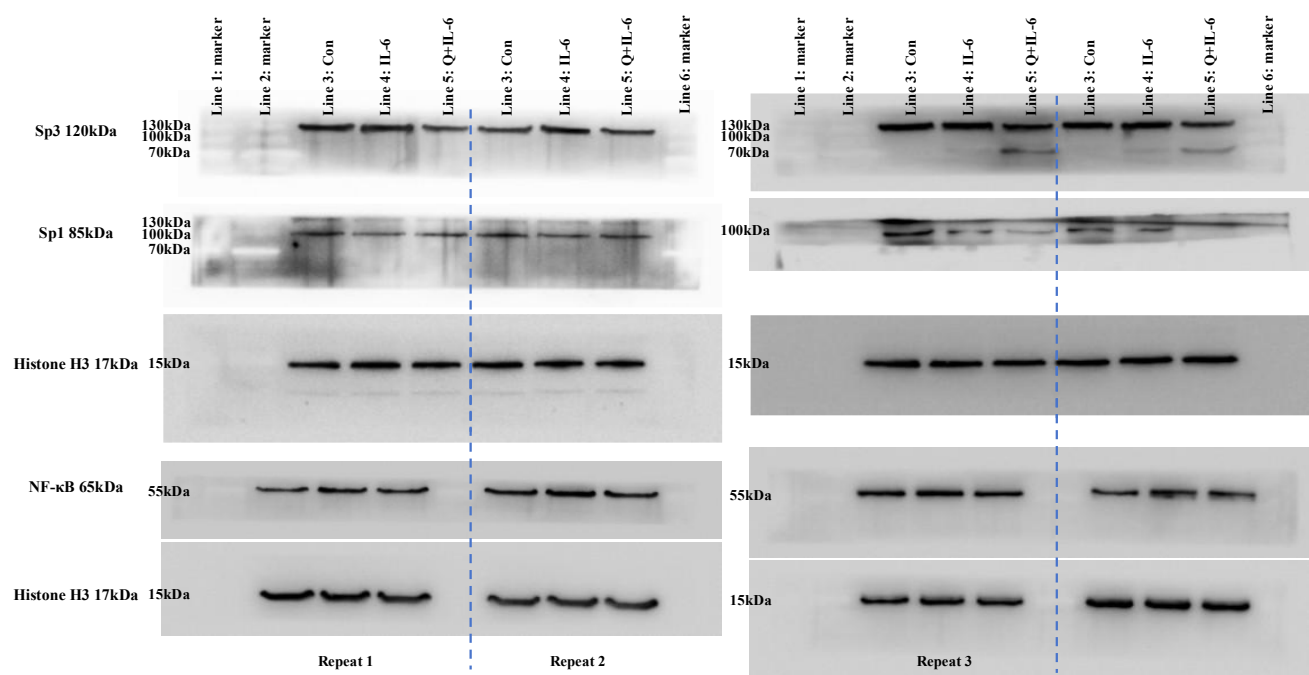

Figure 2L  
Repeat 1

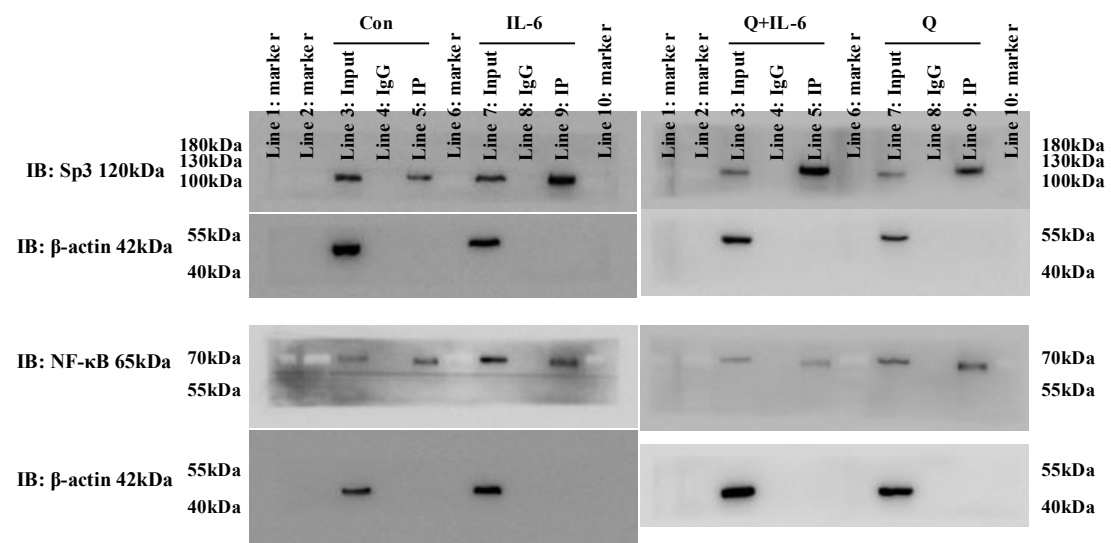

Repeat 2

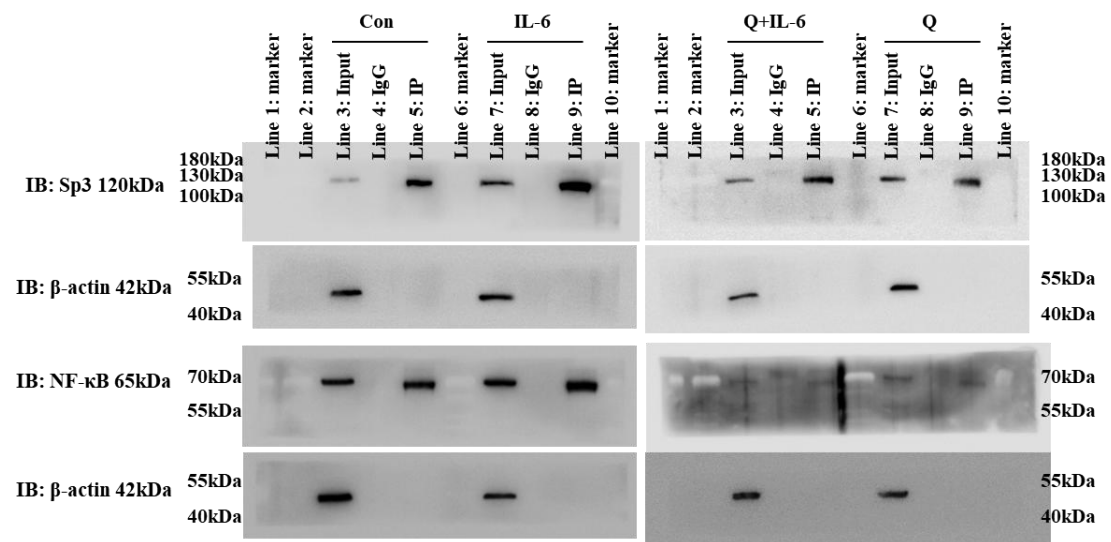

Repeat 3

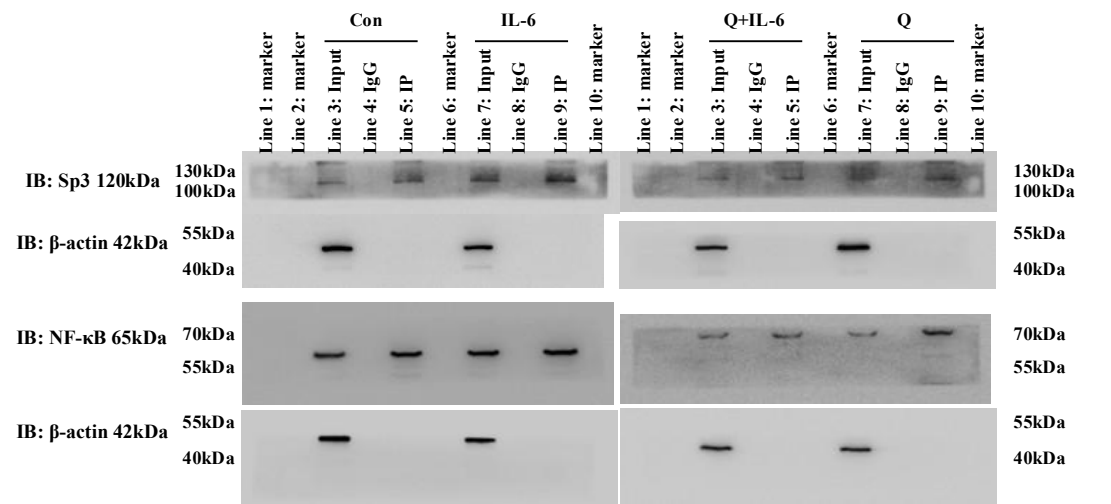

Figure 3A

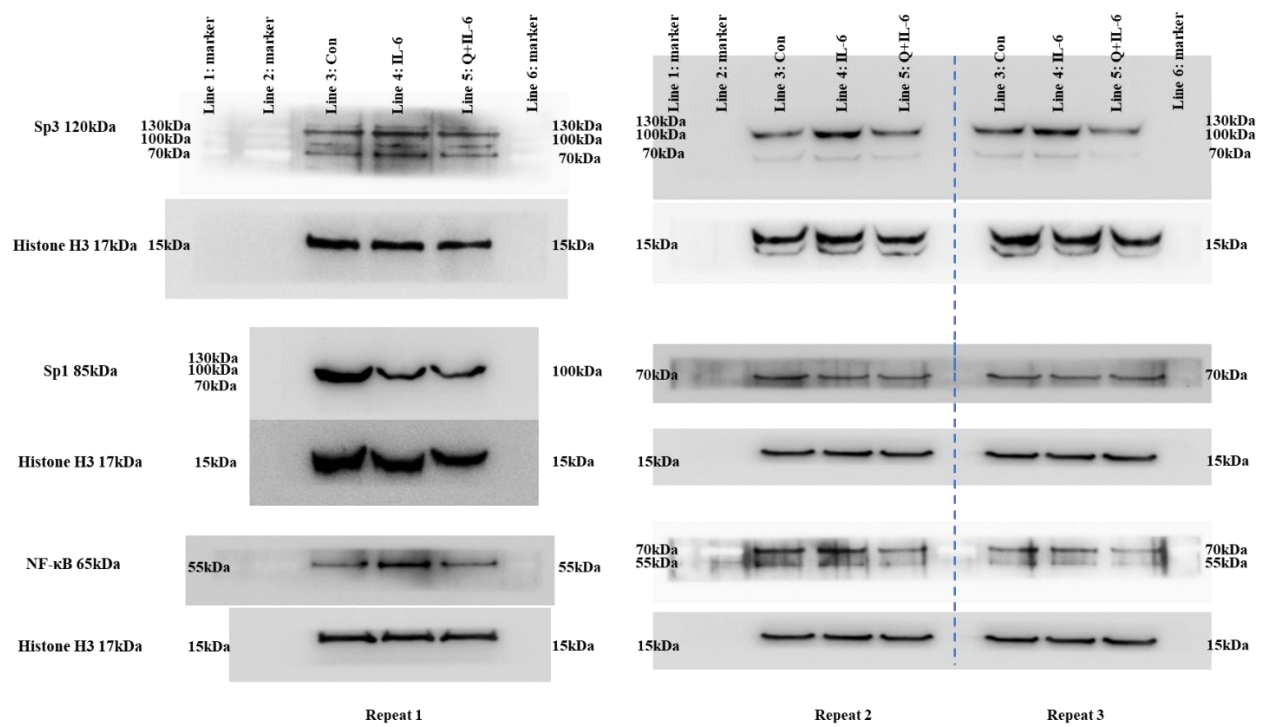

Supplement: Supplementary file 1 — Supplementary Material 1 [file 41598_2026_52669_MOESM1_ESM.pdf]
